# Screening of Salt-Tolerant *Thinopyrum ponticum* Under Two Coastal Region Salinity Stress Levels

**DOI:** 10.3389/fgene.2022.832013

**Published:** 2022-02-04

**Authors:** Chunyan Tong, Guotang Yang, AoenBolige, Terigen, Hongwei Li, Bin Li, Zhensheng Li, Qi Zheng

**Affiliations:** ^1^ State Key Laboratory of Plant Cell and Chromosome Engineering, Institute of Genetics and Developmental Biology, The Innovative Academy of Seed Design, Chinese Academy of Sciences, Beijing, China; ^2^ College of Life Science and Technology, Inner Mongolia Normal University, Hohhot, China; ^3^ University of Chinese Academy of Sciences, Beijing, China

**Keywords:** *Thinopyrum ponticum*, salt tolerance, adult-plant stage, agronomic traits, physiological index, nutrient content

## Abstract

To accelerate the exploitation and use of marginal soils and develop salt-tolerant forage germplasm suitable for the coastal regions of China, seven lines of decaploid tall wheatgrass [*Thinopyrum ponticum* (Podp.) Barkworth and D. R. Dewey, 2*n* = 10*x* = 70] were transplanted under low (.3%) and high (.5%) salt conditions for a comprehensive analysis at the adult-plant stage. Differences were observed among these materials, especially in terms of grass yield, agronomic characteristics, and physiological and biochemical indices. Line C2 grew best with the highest shoot total fresh and dry weights under all conditions except for the milk-ripe stage in Dongying in 2019. The total membership value of C2 also reflected its excellent performance after transplanting. As superior germplasm, its relatively high antioxidant enzyme activities and chlorophyll *a*/*b* ratio suggested C2 may maintain normal metabolic and physiological functions under saline conditions. Furthermore, decaploid tall wheatgrass as a forage grass species has a high nutritive value beneficial for animal husbandry. Accordingly, line C2 may be used as excellent germplasm to develop salt-tolerant cultivars in the Circum-Bohai sea.

## Introduction

Soil salinization is a major environmental stress factor that inhibits normal plant growth while also leading to soil degradation and significant deterioration of the global ecosystem (Bazihizina et al., 2012; Ding et al., 2021). More than 800 million hectares (6.5%) of the land area worldwide contain saline-alkali soil (FAO, 2017; Zhang et al., 2018). In China, the related figure is 100 million, of which over 80% are undeveloped (Xie et al., 2021). Salinized soils, which are mainly distributed in the northwestern, northern, northeastern, and coastal regions of China, result from seawater impregnation, volcanic movement, salt bioaccumulation, uplift of saline groundwater, and agricultural irrigation (Wang et al., 2020). The most effective and environmentally friendly methods for controlling soil salinization involve the absorption, transformation, or transfer of salt from the soil *via* the metabolic and growth activities of plants and microorganisms. Cultivating salt-tolerant crops on salinized land may lead to increased transpiration, decreased groundwater levels, and inhibited soil salinization (Qadir and Oster, 2004). For the past decade, studies on many forage species, such as *Leymus chinensis* (Wang et al., 1994), *Achnatherum splendens* (Xu et al., 2008), and *Hordeum jubatum* L. (Chen et al., 2021), revealed that these species have adapted to salinized soil, suggesting they are useful for controlling and improving salinized soils.

Decaploid tall wheatgrass [*Thinopyrum ponticum* (Podp.) Barkworth and D. R. Dewey, 2*n* = 10*x* = 70, syn. *Agropyron elongatum* (Host) P. Beauv., *Elytrigia pontica* (Podp.) Holub, and *Lophopyrum ponticum* (Podp.) Á Löve] is a perennial forage grass species and an essential wild relative to improving wheat (Shannon, 1978; Sharma et al., 1989). Previous study indicated that it had stronger salt tolerance than most monocotyledonous species, such as rice, wheat, and barley, and some dicotyledonous species, such as alfalfa (Munns and Tester, 2008). Colmer et al. (2006) determined that several *Th. ponticum* accessions could survive a treatment with 750 mM NaCl and some could grow reasonably well at an electrical conductivity of 13.9 dS·m^−1^. Additionally, the enhanced salt tolerance of *Lophopyrum elongatum*, which is an ancestor of *Th. ponticum*, appears to be mediated by genes on chromosomes 2E^e^, 3E^e^, 4E^e^, and 7E^e^ (Dvořák et al., 1988). Furthermore, chromosome 3E^e^ was linked to a 50% decrease in Na^+^ accumulation in the flag leaf (Omielan et al., 1991). Researchers subsequently used the *ph1b* mutant to induce the homoeologous recombination between chromosome 3E^e^ and wheat chromosomes 3A and 3D. An examination of the resulting recombinant lines revealed that the lines with the smallest alien segments exhibited the sodium exclusion trait (Mullan et al., 2009). Moreover, chromosome 5E^b^ of *Th. bessarabicum*, which is another ancestor of *Th. ponticum*, includes at least one major dominant gene for salt tolerance (Forster et al., 1988; King et al., 1997). Thus, *Th. ponticum* chromosomal segments likely carry genes that can be used to increase the salt tolerance of wheat. For example, the dominant salt tolerance gene block in the genome of the wheat-*Th. ponticum* translocation line S148 was detected by a salinity test involving the backcross progenies of S148 and Chinese Spring (Yuan and Tomita, 2015). The saline-tolerant cultivar Shanrong No. 3, which was produced from a somatic hybridization between bread wheat and tall wheatgrass, is a valuable genetic resource for characterizing the mechanisms underlying salt tolerance. Its excellent growth recovery, ionic homeostasis, and ability to excrete toxic products were demonstrated by two-dimensional gel electrophoresis and mass spectrometry analyses (Peng et al., 2009).

The nearly 200,000 ha of saline soil in the Yellow River Delta, which is a representative coastal saline zone in China, are mainly the result of the excessive accumulation of Na^+^ and Cl^−^ ions (Jia et al., 2013). This region has not been well exploited and used, which has seriously hindered the regional economy and ecological development. The salt content of the saline soil widely distributed in the Yellow River Delta can exceed 3% but usually ranges from .6 to 1.0%. Moreover, extreme weather conditions in this area, such as drought in the spring and excessive rainfall in the summer, have been common in recent years. In 2020, Prof. Zhensheng Li proposed a new initiative involving the development of a coastal grass belt, in which salt-tolerant forage grass species are cultivated on 667,000 ha of saline and alkaline soils around the Bohai Sea (Li et al., 2022). Because *Th. ponticum* can grow in saline soil and withstand drought and waterlogging, it may be a suitable forage species for the coastal saline zone in China (Rogers and Bailey, 1963; Shannon, 1978; Weimberg and Shannon, 1988; Jenkins et al., 2010; Guo et al., 2015; Borrajo et al., 2018; Ruf and Emmerling, 2018). Furthermore, the developed root system of tall wheatgrass can accumulate soil organic matter and improve soil fertility, which will positively contribute to regional agricultural and ecological development (Pimentel et al., 1987). To achieve this goal, *Th. ponticum* lines suitable for local conditions must be identified.

In this study, we selected the sexually reproducing plants of seven *Th. ponticum* clone lines as materials for an investigation of their agronomic traits, quality-related characteristics, and physiological and biochemical indices under salt stress conditions. The objective of this study was to identify salt-tolerant *Th. ponticum* germplasm with excellent agronomic characteristics that may be useful for breeding new *Th. ponticum* varieties, ideal for the saline soil in the coastal regions of China.

## Materials and Methods

### Plant Materials

In 2008, Zhensheng Li’s group selected seven phenotypically distinct tall wheatgrass individuals. Their clone lines (designated as C1–C7) were vegetatively propagated by cutting tillers with living roots. The seeds of these clone lines were collected for this study. The clone lines and the seeds were preserved in the laboratory of Zhensheng Li at the Institute of Genetics and Developmental Biology, The Innovative Academy of Seed Design, Chinese Academy of Sciences.

### Study Sites and Field Experiments

The seeds of each clone line were sown in seedling trays containing a mixture of field soil and nutrient soil (3:1). The resulting seedlings with fully expanded second leaves were transplanted on 21 March 2018 to fields at the Haixing Experimental Station of Chinese Academy of Sciences (117.6°E, 38.2°N; .5% soil salt content) and on 9 April 2018 to fields at the Dongying Molecular Design Breeding Experimental Station of Chinese Academy of Sciences (118.9°E, 37.7°N; .3% soil salt content), with three plants per hole. The holes in each row were separated by 30 cm, and the rows were separated by 30 cm. Three replicates of each line were set, and each replicate was grown in a 3.0 m × 1.2 m plot. The transplanted seedlings were irrigated with freshwater to optimize the survival rate. There was no additional artificial irrigation during the cropping season. Plants were cultivated using conventional field management practices. Three uniform plants were selected from each plot, and the plant height (PH, cm), tiller number (TN), spike number (SN), spikelet number per spike (SNPS), leaf fresh weight (LFW, g), stem fresh weight (SFW, g), shoot total fresh weight (STFW, g), and shoot total dry weight (STDW, g) of the *Th. ponticum* plants were analyzed at the flowering and milk-ripe stages in Haixing in 2018 (18HF and 18HM) and Dongying in 2018 (18DF and 18DM) and 2019 (19DF and 19DM). Due to the heavy rain in June 2019, the accuracy of STFW at the flowering stage might be affected. Thus, only STDW was measured in 19DF.

### Analysis of Nutrient Contents

Fresh leaves of three plants were collected from each plot at the flowering stage in Haixing in 2018 and then heated at 105°C for 30 min, dried to a constant weight at 65°C, and ground to a powder and mixed in equal proportions for an analysis of the nutrient content. The dry matter (DM) content was determined after drying samples according to a published method by Zhang (2007). The crude protein (CP) content was determined using the Folin–Ciocalteu reagent (Yang, 1999). The neutral detergent fiber (NDF) content and the acid detergent fiber (ADF) content were measured on the basis of filter bag technology using the ANKOM A2000i automatic and semi-automatic fiber meters, respectively, according to Van Soest et al. (1991) with some modifications. The water-soluble carbohydrate (WSC) content was determined using an anthrone colorimetric technique (Zhang, 2007). The ether extract (EE) content and the ash content were measured as previously described (ISO 6492:1999, IDT and Mlejnkova et al., 2016, respectively), with some modifications. The tannin content (TC) was determined according to the vanillin hydrochloric acid method. The forage relative feeding value (RFV) and total digestible nutrient (TDN) content, as well as the relative forage quality (RFQ), were calculated on the basis of the NDF and ADF data using the following equations (Zhang et al., 2004; Schacht et al., 2010; Xiong et al., 2018):

RFV=dry matter intake(DMI) × digestible dry matter(DDM)/1.29; DMI = 120/NDF; DDM = 88.9 - 0.779 × ADF; TDN = 82.38 - 0.7515 × ADF; RFQ = TDN × DMI/1.23.

### Determination of Physiological and Biochemical Indices

For each replicate, a .05 g fresh sample of three plants was collected at the milk-ripe stage in Dongying in 2019 and used to measure the superoxide dismutase (SOD) activity (absorbance at 560 nm) as described by García-Triana et al. (2010). The catalase (CAT) activity (absorbance at 240 nm) was determined using a method developed by Sima et al. (2011). Chlorophyll *a* (Chl *a*) and chlorophyll *b* (Chl *b*) were extracted using 80% acetone, and their contents were determined by measuring the absorbances of the extracts at 645 and 663 nm as described by Arnon (1949). All absorbances were measured using the SpectraMax190 microplate reader (Molecular Devices, America). The proline (Pro) content was determined using ninhydrin (Bates et al., 1973).

### Data Analysis

The membership function value was used for the total membership value (TMV) analysis. The specific formula is as follows:  X(μ1) = (X − Xmin)/(Xmax − Xmin); X(μ2) = 1 − X(μ1) , where X is a certain measured value for the identified index; μ_1_ is the positive correlation membership function value of each index; μ_2_ is the negative correlation membership function value of each index; X_max_ is the maximum value of this index in all lines; and X_min_ is the minimum value of this index in all lines. The sum of the membership function values for the different indices of each line was calculated, and then the average value was used for ranking. Increases in TMV were associated with increasing plant salt tolerance (Liu et al., 2009; Zhang et al., 2020). Both SPSS17.0 and Excel were used to analyze data (*e.g*., two-sample *t*-test, analysis of variance, and one-way ANOVA analysis). Average values and standard deviations were calculated for all measurements. Figures were prepared using SigmaPlot 12.5.

## Results

### Biomass Indices

**FIGURE 1:**
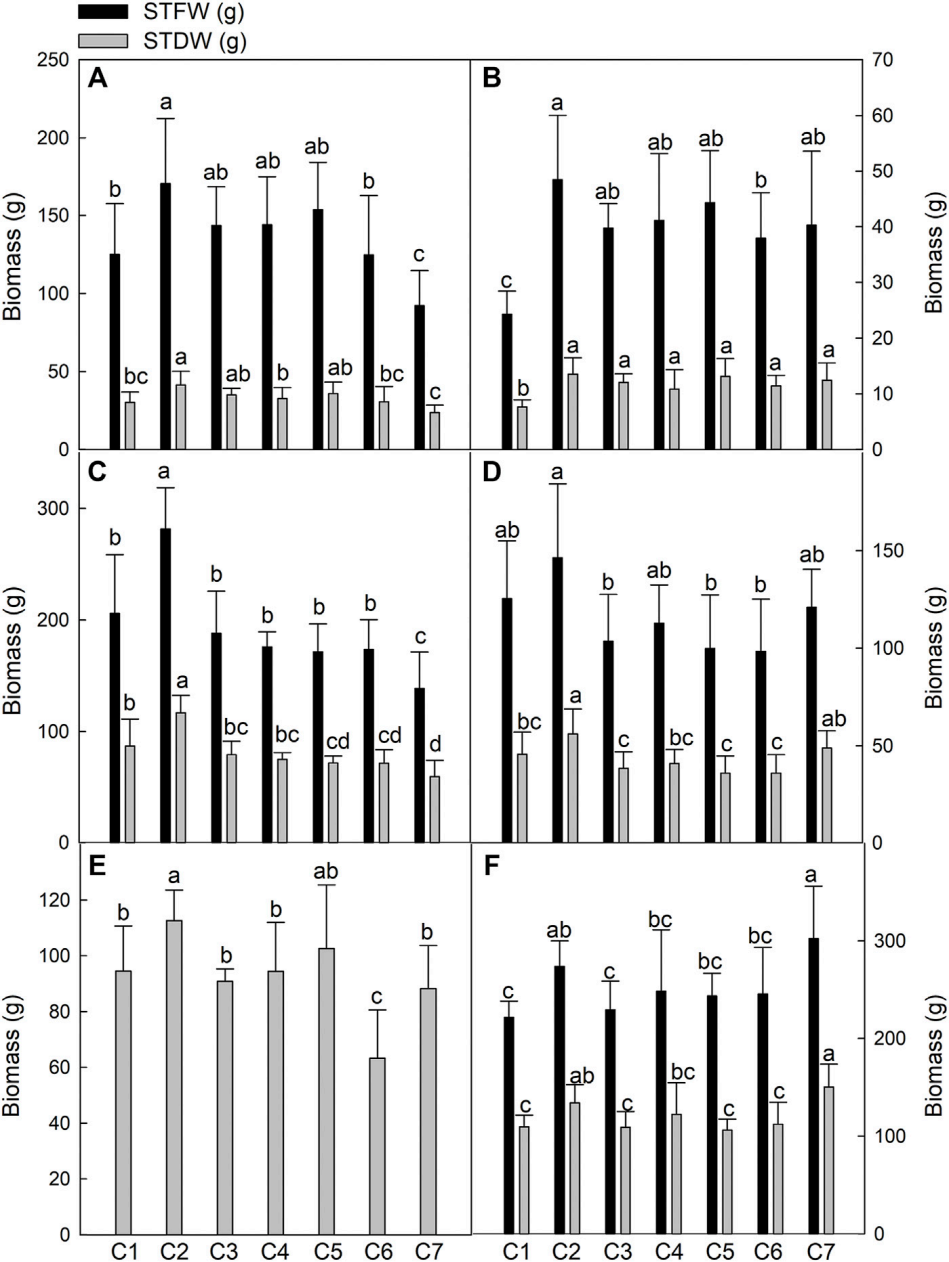
Comparison of biomass of seven *Th. ponticum* lines at the flowering stage at Haixing station in 2018 **(A)**, at the flowering stage at Dongying station in 2018 **(B)**, at the milk-ripe stage at Haixing station in 2018 **(C)**, at the milk-ripe stage at Dongying station in 2018 **(D)**, at the flowering stage at Dongying station in 2019 **(E),** and at the milk-ripe stage at Dongying station in 2019 **(F)**. STFW, shoot total fresh weight; STDW, shoot total dry weight. Different letters indicate a significant difference between seven *Th. ponticum* lines at *p* < .05 level.

To evaluate the growth of seven *Th. ponticum* lines under coastal saline conditions, we analyzed the biomass indices, including STFW and STDW, of each line at the flowering and milk-ripe stages at the Haixing and Dongying experimental stations in 2018 and the Dongying experimental station in 2019. The results for the seven examined lines in six environments are provided in Figure 1. The yields of all *Th. ponticum* lines were generally higher in Haixing than in Dongying during the same growth period in 2018, possibly because the seedlings were transplanted about 20 days earlier in Haixing than in Dongying. In 2018, for most lines, the yields were greater at the milk-ripe stage than at the flowering stage at both experimental stations. A comparison of the data collected during the same developmental period in 2018 and 2019 revealed that most *Th. ponticum* lines produced more biomass in 2019.

During the milk-ripe stage in 2018, STFW was 138.62–281.64 g in Haixing and 98.43–146.40 g in Dongying, whereas STDW was 59.66–116.90 g in Haixing and 36.03–56.13 g in Dongying. In 2019, STDW was 63.26–112.63 g at the flowering stage and 106.33–150.37 g at the milk-ripe stage. Thus, there were distinct biomass differences among the samples. More specifically, STFW and STDW were highest for line C2 under all conditions, except for 19DM. Although the grass yield of line C7 was the highest in 19DM, there were no significant differences between C2 and C7 in terms of STFW and STDW. During the first growing season, the biomass of C2 was significantly higher (*p* < .05) than that of the other lines in 18HM. After one growing season, C2 could still grow vigorously, while C7 flourished similarly at the milk-ripe stage in Dongying in 2019. Compared with the corresponding growth in Dongying, C7 in Haixing had a lower STFW and STDW during both stages in 2018, implying C7 may be suitable for growth in a relatively low salinity soil with high regeneration ability. In summary, the biomass of C2 was prominent among these seven lines under salt stress.

### Agronomic Traits

**FIGURE 2:**
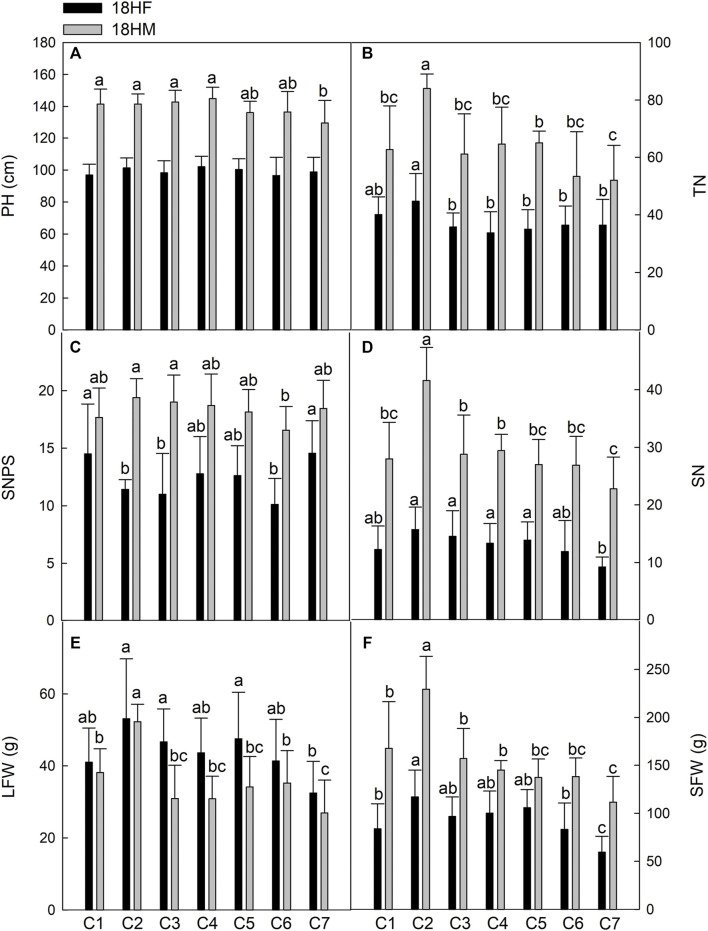
Comparison of main agronomic traits of seven *Th. ponticum* lines at Haixing station at different growing stages. (**A)** Plant height (PH). **(B)** Tiller number (TN). **(C)** Spikelet number per spike (SNPS). **(D)** Spike number (SN). **(E)** Leaf fresh weight (LFW). **(F)** Stem fresh weight (SFW). Different letters indicate a significant difference between seven *Th. ponticum* lines at *p* < .05 level.

**FIGURE 3:**
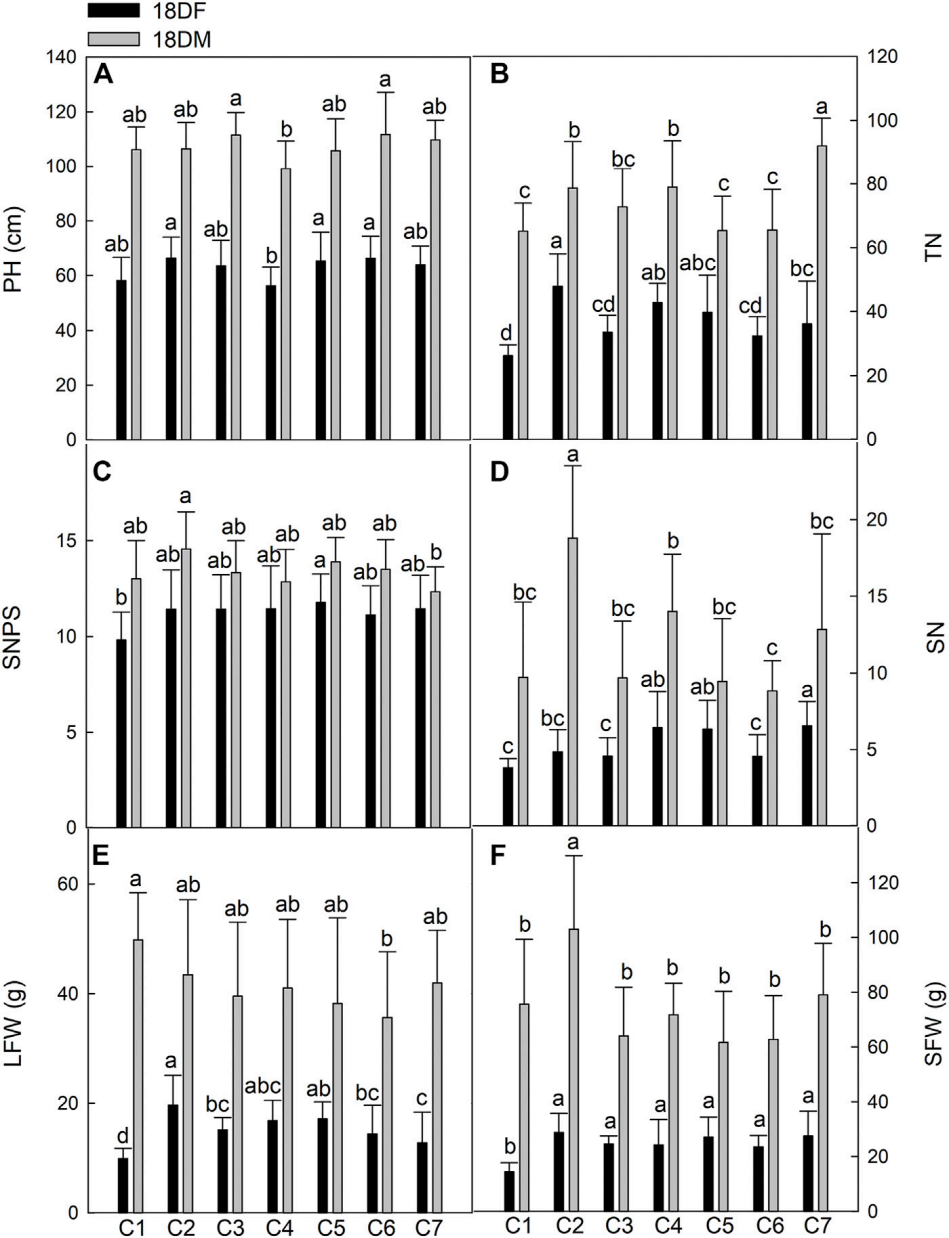
Comparison of main agricultural traits of seven *Th. ponticum* lines at Dongying station during different growth periods. **(A)** Plant height (PH). **(B)** Tiller number (TN). **(C)** Spikelet number per spike (SNPS). **(D)** Spike number (SN). **(E)** Leaf fresh weight (LFW). **(F)** Stem fresh weight (SFW). Different letters indicate a significant difference between seven *Th. ponticum* lines at *p* < .05 level.

Traits related to growth performance, including PH, TN, SNPS, SN, LFW, and SFW, were evaluated for each *Th. ponticum* line in Haixing (Figure 2) and Dongying (Figure 3) in 2018. At the Haixing experimental station, the values of all traits, except for LFW, generally increased as plants matured. The differences among the seven lines were greater in 18HM than in 18HF. For example, there were no significant differences in PH among the seven lines in 18HF. However, in 18HM, the lines were divided into three groups according to their PH, and C7 was significantly shorter (*p* < .05) than C1, C2, C3, and C4. Additionally, PH was the highest for C4, with an average of 144.92 cm, 15.15 cm greater than the PH of C7 (Figure 2A). The SNPS of C2 (19.40) and C3 (19.00) were significantly higher (*p* < .05) than that of C6 (16.56) in 18HM. Line C2 had the highest TN and SN at both stages, and the differences with the corresponding values for the other lines were significant (*p* < .05) in 18HM. In contrast, C7 had the lowest TN in 18HM (Figure 2B) and the lowest SN in 18HF and 18HM (Figure 2D). In Haixing, LFW was a little lower at the milk-ripe stage than at the flowering stage for all lines. At both stages, C2 and C7 had the highest and lowest LFW, respectively. Additionally, C2 had a significantly higher LFW (*p* < .05) than the other lines in 18HM. Similarly, C2 had the highest SFW among the seven lines in both 18HF and 18HM; the differences in SFW between C2 and the other lines were significant (*p* < .05) in 18HM. Considered together, C2 exhibited excellent growth during the first growing season under high salt conditions. At 132 days after transplanting, the three individual plants in each hole of C2 were 142 cm tall, with 84 tillers, 42 spikes, and 19 spikelets, and produced 52.30 g fresh leaves and 229.34 g fresh stems. Moreover, PH, TN, SN, LFW, and SFW were lowest for C7 among the seven examined lines; the differences in these traits between C7 and C2 were significant (*p* < .05). From the flowering stage to the milk-ripe stage, line C2 decreased the minimum LFW and increased the maximum SN, which resulted in its maintaining the fastest growth rate after entering the reproductive growth stage when compared with the other lines.

In Dongying, the values for all of the measured indices increased as *Th. ponticum* plants matured. Line C2 had the highest PH (66.30 cm) at the flowering stage, whereas line C6 had the highest PH (111.68 cm) at the milk-ripe stage. The C4 plants were the shortest at the flowering and milk-ripe stages (56.27 and 99.11 cm, respectively). The TN of C2 (47.84) was significantly higher (*p* < .05) than that of C1, C3, C6, and C7 in 18DF, whereas the TN of C7 (92.00) was significantly higher (*p* < .05) than that of any other line in 18DM. With the exception of C1, all lines had an SNPS greater than 11 in 18DF, with no significant differences between lines. SNPS (11.78) of C5 was the highest in 18DF, whereas SNPS (14.56) of C2 was the highest in 18DM, significantly higher (*p* < .05) than that of C7 (12.33). However, SN was significantly higher (*p* < .05) for C7 (6.56) than for C1, C2, C3, and C6 at the flowering stage, whereas SN was significantly higher (*p* < .05) for C2 (18.78) than for any other line at the milk-ripe stage. LFW of C2 (19.63 g) was significantly higher (*p* < .05) than that of C1, C3, C6, and C7 in 18DF, whereas LFW of C1 (49.77 g) was the highest in 18DM, although there were no significant differences with the other lines, except for C6. Line C2 consistently had the highest SFW at both examined stages, with an average of 28.76 and 102.97 g at the flowering and milk-ripe stages, respectively. There were significant differences (*p* < .05) in SFW between C2 and the other lines at 18DM. In summary, PH, TN, LFW, and SFW of C2 were the highest at the flowering stage, whereas PH of C6; TN of C7; LFW of C1; and SN, SNPS, and SFW of C2 were the highest at the milk-ripe stage. Of the tested lines, C2 consistently grew the fastest. Line C1 initially grew slowly and then grew quickly, whereas C6 exhibited the opposite growth trend.

The total membership values were calculated based on the agronomic performance of the seven *Th. ponticum* lines in the four environments of 2018. There were substantial differences in the analyzed traits among the seven lines, which resulted in a wide range of TMV (Table 1). In Haixing, based on the size of TMV, the rank order (highest to lowest) of the membership functions was C2, C5, C4, C3, C1, C6, and C7 in 18HF, whereas it was C2, C1, C3, C4, C5, C6, and C7 in 18HM. In Dongying, the rank order (highest to lowest) of TMV was C2, C5, C7, C4, C3, C6, and C1 at the flowering stage, whereas it was C2, C7, C1, C4, C3, C6, and C5 at the milk-ripe stage. Regardless of the condition, C2 grew better than the other lines at the adult-plant stage. Its scores (.89 in 18HF and .97 in 18HM) were far higher than those of the second-best line (.64 in 18HF and .46 in 18HM) under high salt stress. Line C7 ranked third (.72 in 18DF) and second (.53 in 18DM) in Dongying but exhibited the poorest growth in Haixing, suggesting it is appropriate for soil with a salt content less than .3% (*i.e*., it is sensitive to high salinity).

**TABLE 1:** Membership function analyses of salt tolerance in seven *Th. ponticum* lines.

Stages	Lines	PH	TN	SNPS	SN	LFW	SFW	STFW	STDW	TMV	Rank
18HF	C1	.05	.58	.99	.47	.42	.42	.42	.37	.46	5
	C2	.86	1.00	.30	1.00	1.00	1.00	1.00	1.00	.89	1
	C3	.30	.18	.20	.82	.69	.65	.66	.64	.52	4
	C4	1.00	.00	.60	.63	.54	.71	.66	.51	.58	3
	C5	.70	.11	.57	.72	.73	.80	.79	.69	.64	2
	C6	.00	.24	.00	.41	.43	.41	.42	.39	.29	6
	C7	.41	.24	1.00	.00	.00	.00	.00	.00	.21	7
18HM	C1	.77	.33	.39	.28	.44	.48	.47	.48	.46	2
	C2	.78	1.00	1.00	1.00	1.00	1.00	1.00	1.00	.97	1
	C3	.86	.28	.86	.32	.16	.39	.35	.34	.44	3
	C4	1.00	.39	.76	.35	.15	.28	.26	.27	.43	4
	C5	.43	.41	.55	.22	.28	.22	.23	.21	.32	5
	C6	.45	.04	.00	.22	.33	.23	.24	.21	.21	6
	C7	.00	.00	.66	.00	.00	.00	.00	.00	.08	7
18DF	C1	.18	.00	.00	.00	.00	.00	.00	.00	.02	7
	C2	1.00	1.00	.82	.38	1.00	1.00	1.00	1.00	.90	1
	C3	.73	.34	.82	.28	.54	.71	.64	.75	.60	5
	C4	.00	.77	.83	.96	.71	.69	.70	.55	.65	4
	C5	.90	.63	1.00	.92	.75	.89	.83	.93	.85	2
	C6	.99	.29	.66	.27	.46	.64	.56	.64	.56	6
	C7	.76	.46	.83	1.00	.30	.91	.66	.81	.72	3
18DM	C1	.56	.00	.30	.09	1.00	.34	.56	.48	.42	3
	C2	.58	.50	1.00	1.00	.55	1.00	1.00	1.00	.83	1
	C3	.98	.28	.45	.08	.27	.06	.11	.12	.30	5
	C4	.00	.52	.24	.52	.38	.24	.30	.25	.31	4
	C5	.53	.01	.70	.06	.18	.00	.03	.00	.19	7
	C6	1.00	.01	.53	.00	.00	.03	.00	.00	.20	6
	C7	.85	1.00	.00	.40	.45	.42	.47	.64	.53	2

Annotation: 18HF and 18HM mean investigation at the flowering and milk-ripe stages in Haixing in 2018, respectively. 18DF and 18DM indicate investigation at the flowering and milk-ripe stages in Dongying in 2018, respectively. PH, plant height; TN, tiller number; SNPS, spikelet number per spike; SN, spikelet number; LFW, leaf fresh weight; STW, stem fresh weight; STFW, shoot total fresh weight; STDW, shoot total dry weight; TMV, total membership value.

### Nutrient Indices

The results of the analysis of eight nutrient indices in *Th. ponticum* lines are provided in Table 2. There were no significant differences among the lines for the following six indices: DM (39.17%–42.85%FW), CP (13.15%–14.45%DM), NDF (53.21%–56.30%DM), ADF (28.18%–30.24%DM), EE (3.42%–3.86%DM), and WSC (1.88%–2.39%DM). However, there were significant variations in TC and the ash content. Specifically, TC was significantly higher (*p* < .05) in C2 (2.29 mg/g FW) than in any other line. The ash content was the highest in C5 (9.75%DM) and then C1 (9.69%DM), whereas it was the lowest in C6 (8.46%DM).

**TABLE 2:** Comparison of nutrient content for seven *Th. ponticum* lines at the adult-plant stage.

Lines	Dry matter (%FW)	Curd protein (%DM)	Neutral detergent fiber (%DM)	Acid detergent fiber (%DM)	Ether extract (%DM)	Water-soluble carbohydrates (%DM)	Tannin content (mg/g FW)	Ash (%DM)
C1	40.66 ± .26	13.15 ± .63	56.30 ± 3.12	29.86 ± .86	3.57 ± .16	1.88 ± .17	2.00 ± .18^cd^	9.69 ± .76^a^
C2	39.17 ± 1.77	13.37 ± 1.05	56.21 ± .40	30.24 ± .61	3.44 ± .12	1.88 ± .03	2.29 ± .11^a^	9.14 ± .59^ab^
C3	42.59 ± 4.29	14.01 ± .41	54.80 ± 1.78	29.21 ± .78	3.67 ± .24	2.07 ± .31	1.51 ± .07^f^	8.98 ± .32^ab^
C4	41.65 ± 3.42	14.45 ± .17	55.06 ± 3.22	29.46 ± 1.24	3.42 ± .85	2.30 ± .45	1.92 ± .04^d^	8.87 ± .55^ab^
C5	41.49 ± 4.41	14.23 ± .41	54.11 ± 1.27	29.12 ± .75	3.49 ± 1.49	2.06 ± .41	2.13 ± .09^b^	9.75 ± .70^a^
C6	40.10 ± 1.13	13.86 ± .83	53.21 ± 3.01	28.18 ± 2.13	3.86 ± .85	2.04 ± .18	1.69 ± .04^e^	8.46 ± .19^b^
C7	42.85 ± 3.38	14.38 ± .87	54.55 ± 2.95	28.61 ± .99	3.45 ± 1.16	2.39 ± .46	2.10 ± .08^bc^	8.57 ± .55^b^

Annotation: different letters indicate a significant difference between seven *Th. ponticum* lines at *p* < .05 level

The NDF and ADF data were used to calculate the RFV, TDN, and RFQ of the *Th. ponticum* lines to assess the forage quality and value. There were no significant differences in the RFV (108–118), TDN (59–62), and RFQ (103–113). In short, nutrient indices expressed no significant differences among the lines except for TC and ash.

### Physiological and Biochemical Indices

**FIGURE 4:**
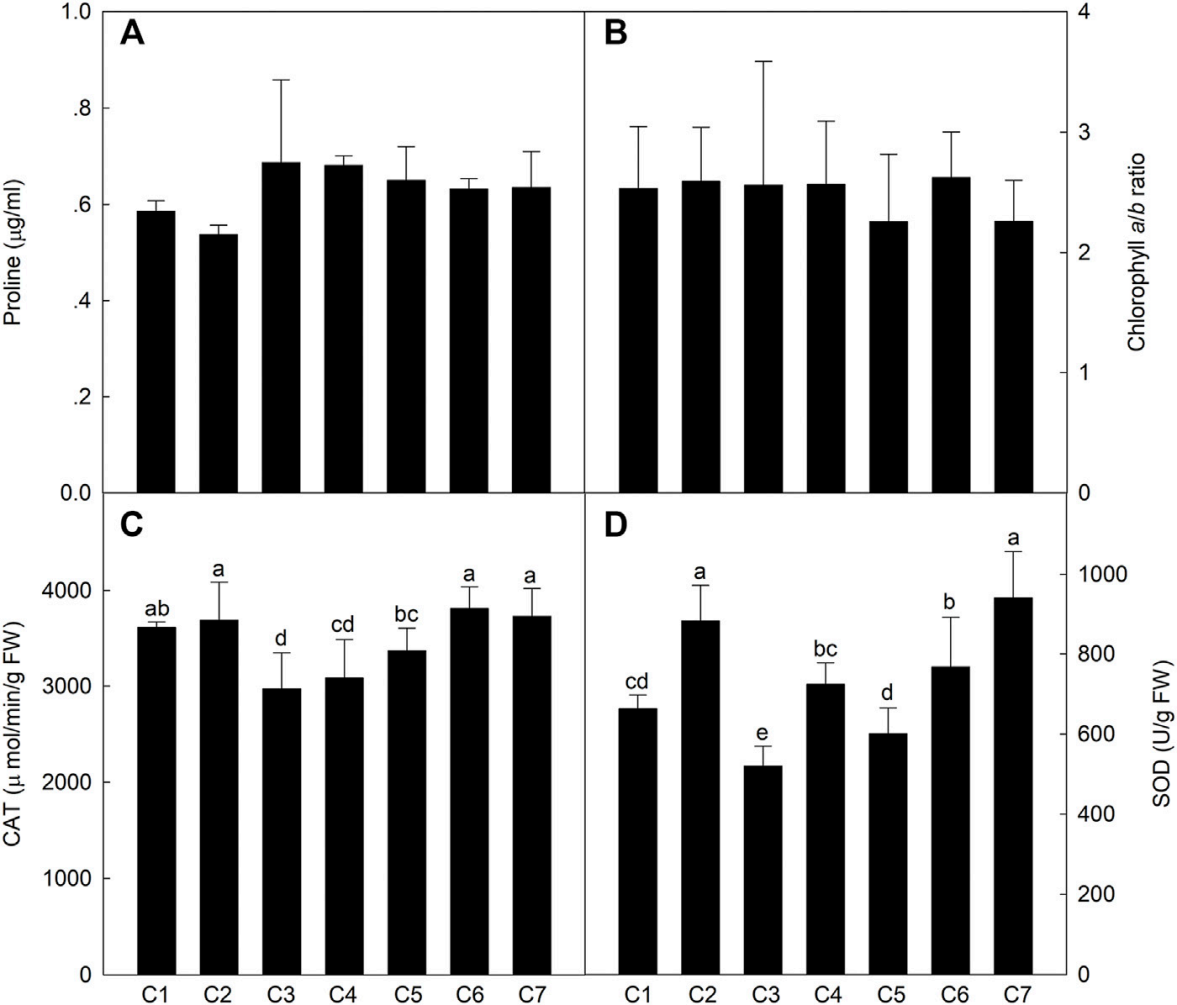
Comparison of the content of proline **(A)**, chlorophyll *a*/*b* ratio **(B)**, activities of CAT **(C),** and SOD **(D)** in the seven *Th. ponticum* lines. Different letters indicate a significant difference between seven *Th. ponticum* lines at *p* < .05 level.

To compare the physiological and biochemical indices of the different lines exposed to saline conditions, osmotic regulatory compounds and protective enzymes were analyzed in the leaves at the milk-ripe stage. The leaf Pro content was .54–.69 μg/ml (Figure 4), with no significant differences among the *Th. ponticum* lines. There were also no significant differences in the Chl *a*/*b* ratio. However, the Chl *a*/*b* ratio was the highest for C6 (2.62) and then C2 (2.59), whereas it was the lowest for C5 and C7 (2.26). The CAT activity of C1, C2, C6, and C7 were higher levels in these materials, and there was no significant difference between these lines. The SOD activity was the highest in C7 and C2, with no significant difference. In general, *Th. ponticum* can maintain normal physiological metabolism under salt stress by increasing its main osmotic regulation substances, especially line C2.

## Discussion

### A Comprehensive Analysis Is Necessary to Assess Forage Salt Tolerance

Plant salt tolerance is a comprehensive trait involving multiple signaling pathways (Strizhov et al., 1997; Gohari et al., 2021). Because of the decrease in water potential and increase in osmotic pressure associated with saline soils, plant growth and development are seriously hindered by a decrease in the absorption of water by the roots, the closing of stomata, an increase in the leaf temperature, the inhibited differentiation of tissues and organs, and delayed cells growth. Salt stress can also severely affect plant physiological processes (Munns and Tester, 2008; Roy et al., 2014). Two new salt-tolerant bread wheat cultivars (“Maycan” and “Yildiz”) have significantly higher Chl *a*, Chl *b*, and total chlorophyll contents than salt-sensitive wheat varieties under saline conditions (Aycan et al., 2021). In response to salt stress, plants accumulate small organic compounds, such as Pro, to resist the adverse effects of stress while also synthesizing antioxidant enzymes to scavenge excess reactive oxygen species. As the core enzymes in the antioxidant system, SOD and CAT can minimize plant damage caused by reactive oxygen species by degrading hydrogen peroxide under saline conditions (Sekmen et al., 2007). Gohari et al. (2021) determined that, in grapevine, SOD and CAT activities increased following exposure to salinity stress. Additionally, Pro is an important component under salt treatment of plants, whose function may protect protein turnover normally and upregulate stress-protective proteins in the desert plant *Pancratium maritimum* L. (Khedr et al., 2003). Kim et al. (2016) performed Pro analyses of 46 switchgrass under salt stress, and the result revealed that salt-sensitive lines increased sharply. However, salt-resistant lines increased slightly in Pro concentration in response to salt treatment. In our study, C2 had the lowest Pro content, stable biomass advantage, and relatively high CAT and SOD activities and Chl *a*/*b* ratio, suggesting it may be better able to maintain normal metabolic and physiological activities under salt stress than the other lines.

Because plant salt tolerance varies among growth stages, it is difficult to accurately assess using a single index (An et al., 2021). To the best of our knowledge, herbage salt tolerance is currently evaluated primarily on the basis of the biomass, K^+^ and Na^+^ contents, and the relative water content during the germination or seedling stage (Johnson, 1991; Sagers et al., 2017). There has been relatively little research on herbage salt tolerance at the adult-plant stage, especially during the flowering and mike-ripe stages. To evaluate plant salt tolerance, a comprehensive analysis of morphological, physiological, biochemical, and other indices should be conducted. In the current study, the agronomic performance of C2 was generally the best among the *Th. ponticum* lines transplanted in coastal saline soil (.3% or .5% salinity). In Haixing, TN, SN, LFW, and SFW were the highest for C2 at two sampling stages. In Dongying, SNPS, SN, and SFW were the highest for C2 at the milk-ripe stage. A membership function analysis revealed that C2 exhibited better overall growth and produced more seeds than the other examined lines in both experimental fields. Additionally, STFW and STDW were higher for C2 than for the other six tall wheatgrass materials in six different environments, implying that C2 has a greater production potential under salt stress conditions than the other lines.

### Importance of the Nutrient Content for Evaluating Forage Species

The nutrient content directly affects animal feed intake and forage palatability. The nutritional value of herbage is generally evaluated according to CP and the crude fiber (CF) content. Van Soest et al. (1991) divided CF into NDF, ADF, and acid detergent lignin, whose contents can directly affect animal feed intake and digestibility. For example, sheep feed intake decreased as NDF and ADF of perennial ryegrass (*Lolium perenne*) increased (Aitchison et al., 1986). Moreover, a high NDF in silage corn can decrease beef cattle feed intake (Tjardes et al., 2002). The CP content, which comprises protein and non-protein nitrogenous compounds, represents the essential materials for animal tissues, cells, and metabolism. An examination of the forage yield and quality traits of a collection of 50 tall wheatgrass accessions from the USDA-ARS Western Regional Plant Introduction Station demonstrated that the shoot CP ranged from 6.6 to 11% at the heading stage (Vogel and Moore, 1998). Additionally, dynamic changes were observed in forage grass CP during different growth periods. On the basis of their data for 17 *Th. ponticum* accessions from Iran, Jafari et al. (2014) revealed the average CP contents were 7.96 and 4.86% for two and one cutting managements, respectively. In the current study, the average NDF and ADF of seven tall wheatgrass lines were 54.89%DM and 29.24%DM, respectively. Furthermore, the CP of each line, which exceeded 13%DM, accounted for a higher percentage of the DM than the CP of *Poa annua* L. (9.93%) (Qu and Shi, 2017), silage corn (7.68%), and other gramineous forage species (Liu et al., 2018). Thus, *Th. ponticum* lines are useful as high-quality protein feed.

An increase in NDF and a decrease in CP will lead to an increase in TC. Some studies indicated that the bitter taste associated with a tannin DM greater than 1% would substantially decrease plant palatability. However, when TC is less than 1%, the accumulation of tannin may contribute to an increase in protein conversions and prevent ruminal tympany among herbivores (Barry and McNabb, 1999). In the present study, the TC of C2 (2.29 mg/g FW, equivalent to .45%DM) was within the relative permissible TC range and was significantly higher (*p* < .05) than the TC of the other lines, implying that C2 is a palatable line beneficial for nutrient metabolism and the prevention of bloating.

Hay quality is mainly assessed via a comprehensive analysis that divides the total digestible nutrients of forage materials on the basis of detergent fiber and DMI. As indices used for evaluating quality, TDN, RFV, and RFQ are positively correlated with forage palatability and digestibility (Rohweder et al., 1978). Xiong et al. (2018) analyzed the nutrient content of *Avena sativa* L. in different regions in China and determined that the TDN, RFV, and RFQ of *A. sativa* were, respectively, 52, 93, and 88 in Shanxi province and 51, 86, and 81 in Gansu province. In this study, the average TDN, RFV, and RFQ values of *Th. ponticum* were 60.41, 112.31, and 107.61, respectively, which were higher than the corresponding values for *A. sativa*. The WSC and EE values of *Th. ponticum* were 2.09%DM and 3.56%DM, respectively, representing a relatively large proportion of DM. The EE of this study was greater than the EE reported for some gramineous species, including oat (2.65%), *Setaria viridis* (L.) Beauv (2.11%), and *Sorghum–*sudangrass hybrids (1.26%) (Liu et al., 2018). These findings suggest that *Th. ponticum* growing in the Circum-Bohai sea region is a forage grass species with a high nutritive value, with implications for animal husbandry.

### Decaploid Tall Wheatgrass Can Accelerate the Improvement of Coastal Saline Soils

*Elytrigia* (now named *Thinopyrum*), which is a genus in the gramineous wheat subfamily, includes the following species: *Elytrigia repens*, *Elytrigia smithii*, *Elytrigia trichophora*, *Elytrigia elongata*, and *Elytrigia intermedia* (Geng, 1959). Many studies have confirmed the differential salt tolerance of *Elytrigia* varieties (Dewey, 1960; Shannon, 1978). *Th. ponticum*, which is usually referred to as decaploid tall wheatgrass, has been used as a wild relative for the genetic improvement of wheat stress resistance (Li et al., 2008; Wang, 2011).

There are only a few reports that describe *Th. ponticum* as a high-quality pasture crop with strong tolerance to stress conditions. Although 25 *Th. ponticum* materials were selected and compared as early as in 1960 (Dewey, 1960), relatively few *Th. ponticum* varieties are available for production (Vogel and Moore, 1998). To date, eight *Th. ponticum* varieties (Jose, Largo, Alkar, Nebraska98526, Orbit, Platte, NFTW6001, and Plainsmen) have been developed and released in the United States and Canada (Alderson and Sharp, 1994; Trammell et al., 2016; Trammell et al., 2021). Two salt-tolerant varieties (Tyrrell and Dundas) were bred in Australia (Rogers and Bailey, 1963; Smith and Kelman, 2000). In China, *Th. ponticum* was initially transported from the United States to the Tianshui Experimental Station of Soil and Water Conservation (Gansu province). Professor Zhensheng Li brought it to the Laboratory of Genetics and Plant Breeding of CAS in Beijing in 1954 and then to Northwest Institute of Botany (Yangling, Shaanxi province) in 1956 to be used for wheat distant hybridizations. There are no decaploid tall wheatgrass varieties that have been certified in China, which has seriously restricted their use. The Yellow River Delta has many undeveloped land resources that are suitable for the cultivation of salt-tolerant herbage. Because of its excellent tolerance to salt, waterlogging, and drought stresses, *Th. ponticum* is an ideal forage species for improving saline coastal soils and developing animal husbandry. Line C2, which was identified as a superior germplasm in the present study, may be used to develop improved cultivars with elite traits resulting from the cross pollination between C2 individuals or between C2 and other appropriate lines with high regeneration ability and accelerate the application of its useful genes in improving wheat genetic variability by chromosome engineering. Furthermore, these materials would achieve the target of planting 667,000 ha salt-tolerant forage grass in saline and alkaline soils in the Circum-Bohai sea region.

## Conclusion

On the basis of its high biomass and excellent agronomic performances revealed in this study, C2 is better suited for saline environments than the other examined tall wheatgrass lines. Thus, it may be relevant for optimizing the use of marginal soils and developing improved *Th. ponticum* cultivars that can be planted in the Circum-Bohai sea region of China.

## Data Availability

The raw data supporting the conclusion of this article will be made available by the authors without undue reservation.

## Glossary

ADF: acid detergent fiber
CAT: catalase
CF: crude fiber
Chl *a*: chlorophyll *a*
Chl *b*: chlorophyll *b*
CP: crude protein
DDM: digestible dry matter
DM: dry matter
DMI: dry matter intake
EE: ether extract
LFW: leaf fresh weight
NDF: neutral detergent fiber
PH: plant height
Pro: proline
RFQ: relative forage quality
RFV: relative feeding value
SFW: stem fresh weight
SN: spike number
SNPS: spikelet number per spike
SOD: superoxide dismutase
STDW: shoot total dry weight
STFW: shoot total fresh weight
TC: tannin content
TDN: total digestible nutrient
TMV: total membership value
TN: tiller number
WSC: water-soluble carbohydrate
18DF: 2018 Dongying, flowering stage
18DM: 2018 Dongying, milk-ripe stage
18HF: 2018 Haixing, flowering stage
18HM: 2018 Haixing, milk-ripe stage
19DF: 2019 Dongying, flowering stage
19DM: 2019 Dongying, milk-ripe stage
